# H11-induced immunoprotection is predominantly linked to N-glycan moieties during *Haemonchus contortus* infection

**DOI:** 10.3389/fimmu.2022.1034820

**Published:** 2022-10-25

**Authors:** Chunqun Wang, Lu Liu, Tianjiao Wang, Xin Liu, Wenjie Peng, Ratnesh Kumar Srivastav, Xing-Quan Zhu, Nishith Gupta, Robin B. Gasser, Min Hu

**Affiliations:** ^1^ State Key Laboratory of Agricultural Microbiology, College of Veterinary Medicine, Huazhong Agricultural University, Wuhan, China; ^2^ College of Life Science and Technology, Huazhong University of Science and Technology, Wuhan, China; ^3^ Shanghai Center for Systems Biomedicine, Shanghai Jiao Tong University, Shanghai, China; ^4^ Department of Biological Sciences, Birla Institute of Technology and Science – Pilani (BITS-P), Hyderabad, India; ^5^ College of Veterinary Medicine, Shanxi Agricultural University, Jinzhong, China; ^6^ Department of Molecular Parasitology, Faculty of Life Sciences, Humboldt University, Berlin, Germany; ^7^ Melbourne Veterinary School, The University of Melbourne, Parkville, VIC, Australia

**Keywords:** parasitic nematode, vaccine, H11 antigen, N-glycosylation, N-glycan, IgG antibodies, immunoprotection

## Abstract

Nematodes are one of the largest groups of animals on the planet. Many of them are major pathogens of humans, animals and plants, and cause destructive diseases and socioeconomic losses worldwide. Despite their adverse impacts on human health and agriculture, nematodes can be challenging to control, because anthelmintic treatments do not prevent re-infection, and excessive treatment has led to widespread drug resistance in nematode populations. Indeed, many nematode species of livestock animals have become resistant to almost all classes of anthelmintics used. Most efforts to develop commercial anti-nematode vaccines (native or recombinant) for use in animals and humans have not succeeded, although one effective (dead) vaccine (Barbervax) has been developed to protect animals against one of the most pathogenic parasites of livestock animals – *Haemonchus contortus* (the barber’s pole worm). This vaccine contains native molecules, called H11 and H-Gal-GP, derived from the intestine of this blood-feeding worm. In its native form, H11 alone consistently induces high levels (75-95%) of immunoprotection in animals against disease (haemonchosis), but recombinant forms thereof do not. Here, to test the hypothesis that post-translational modification (glycosylation) of H11 plays a crucial role in achieving such high immunoprotection, we explored the N-glycoproteome and N-glycome of H11 using the high-resolution mass spectrometry and assessed the roles of N-glycosylation in protective immunity against *H. contortus*. Our results showed conclusively that N-glycan moieties on H11 are the dominant immunogens, which induce high IgG serum antibody levels in immunised animals, and that anti-H11 IgG antibodies can confer specific, passive immunity in naïve animals. This work provides the first detailed account of the relevance and role of protein glycosylation in protective immunity against a parasitic nematode, with important implications for the design of vaccines against metazoan parasites.

## Introduction

Roundworms (nematodes) represent one of the largest groups of animals (phylum Nematoda). While most nematodes are free-living, many of them are major pathogens of plants and animals worldwide (1). Nematode diseases (nematodiases) of plants cause major production losses, equating to ~ US$ 80 billion per annum, and the adverse economic impact of nematodiases on agricultural animals is estimated at ~ US$ 30 billion worldwide. Humans are also affected by a range of nematodes, such as soil-transmitted helminths (STHs), filarioids and food-borne worms, which cause neglected diseases, imposing a global burden of ~10 million disability-adjusted life years (DALYs) per annum (2, 3). In spite of their very substantial, often chronic impact, nematodes can be very challenging to control, because anthelmintic treatments do not prevent re-infection, and repeated treatments lead to genetic resistance in nematode populations (4, 5). Indeed, many nematode species of livestock animals have become resistant to almost all of the main classes of anthelmintics (or pesticides) sold commercially.

To circumvent the anthelmintic resistance problem, there have been major efforts to develop anti-nematode vaccines (native or recombinant) for use in animals and humans (6, 7). However, the majority of these efforts have not been successful due to limited insight into the protective epitopes and underlying immune response. Presently, only one effective (dead) anti-nematode vaccine (called Barbervax) has been developed and commercialised that protects livestock animals against the disease (= haemonchosis) caused by *Haemonchus contortus* (the barber’s pole worm). This vaccine comprises native molecules and molecular complexes, including H11 (8) and H-Gal-GP (9), derived from the intestinal tract of this worm. Although native H11 consistently achieves high levels (75-95%) of protection (8, 10, 11), the substantial efforts attempting to produce recombinant forms of some of these molecules, particularly H11, in an academic or commercial context were consistently unsuccessful (12, 13). Also attempts to develop a well-defined, recombinant vaccine molecules, employing a range of bacterial (14), yeast (15), insect cell (16) and *Caenorhabditis elegans* expression systems (17, 18), have not been successful.

Although H11 has remained a prime vaccine molecule candidate for more than three decades, there has been no detailed investigation of the reason(s) why recombinant proteins expressed in prokaryotic and eukaryotic organisms have failed to protect at levels achieved using native H11. Here, we hypothesise that post-translational modification(s) play a crucial role in immunoprotection against *H. contortus* – which represents a powerful model system for parasitic nematodes more generally (19). A critical appraisal of current literature reveals evidence that glycosylation can associate with immunogenicity and/or antigenicity in hosts infected with particular nematodes (e.g., *Dictyocaulus*) (20), but detailed information is very scant. In other host-pathogen systems, N-glycosylation has been associated with the immunogenicity of proteins and immune modulation (21–23), thereby elevating the relevance of using glycomic and glycoproteomic tools to discover the role of carbohydrate antigens in inducing immune responses (24–27). Despite these developments, no detailed information is available on parasitic nematodes.

Here, we explore the N-glycoproteome and N-glycome of H11 using high-resolution mass spectrometry and assess the roles of N-glycosylation in protective immunity against *H. contortus*. This work provides the first detailed insight into the relevance and role of protein glycosylation in protective immunity against a parasitic nematode. It should have important implications for developing vaccines against metazoan parasites and understanding host immune responses to these parasites.

## Materials and methods

### Parasite materials

Different developmental stages of *H. contortus* were maintained, collected, processed and stored using well-established protocols (28, 29). Specifically, infective third-stage larvae (iL3s, Haecon-5 strain) were obtained from coproculture (28), isolated and maintained at 15 ˚C. Exsheathed L3s (xL3s) were produced by incubation with 0.15% (v/v) sodium hypochlorite at 37°C for 10 min (29). Adult *H. contortus* were obtained from the abomasa of infected goats.

### H11 antigen

Native H11 was isolated from adult *H. contortus* using concanavalin A-sepharose (GE Healthcare), as previously described (8). Briefly, 15 g of the worms were homogenized in ice-cold phosphate-buffered saline (PBS, pH 7.4) for 25 min using a glass homogenizer. The homogenate was centrifuged (12,000 *g* for 25 min) and the pellet was extracted four times with 1% (v/v) Thesit in PBS, and filtered (0.45 µM). H11 was isolated using concanavalin A-sepharose columns. The column-bound H11 was washed 3 times (20 mM Tris-HCl), followed by elution using a buffer containing 200 mM of methyl-D-mannopyranoside and methyl-D-glucopyranoside. The resultant solution was enriched and filtered (0.22 µM). This final filtrate was designated the native H11 antigen. Protein concentration was estimated using a bicinchoninic acid assay (BCA) kit (Beyotime Biotechnology), and quality was assessed by SDS-PAGE analysis and Coomassie blue staining.

### N-glycoproteome analysis

Total protein (400 μg) was reduced with 5 mM dithiothreitol and then alkylated with 11 mM iodoacetamide. The reduced protein sample was digested with trypsin at an enzyme-to-protein ratio of 1:50. After tryptic digestion, the hydrophilic interaction chromatography (HILIC) enrichment of N-glycopeptides was carried out as reported previously (30). Glycopeptides were subjected to de-glycosylation by PNGase F and PNGase A (31). Briefly, 2 μL of PNGase F in 50 μL of 50 mM NH_4_HCO_3_ buffer was incubated at 37°C overnight. The remaining peptides were subjected to HILIC enrichment and reconstituted in 2 μL of PNGase A in 50 μL of 50 mM sodium acetate solution overnight at 37°C. Then PNGase F- and A-deglycosylated peptides were acidified with formic acid, purified using an EASY-nLC 1000 UPLC system and subjected to liquid chromatography-tandem mass spectrometry (LC-MS/MS) analysis using an Orbitrap Fusion™ Tribrid™ instrument (Thermo Fisher Scientific, Bremen, Germany).

MS data were analysed using the software package MaxQuant to identify peptides, followed by searches against the UniProtKB *H. contortus* database (24,277 proteins), concatenated using the reverse decoy database. Glycosylation sites were inferred using the consensus of two biological replicates. Amino acid sequence motifs comprising at least 20 residues (+/-10) of the N-glycosylation sites were analysed using the MoMo program (https://meme-suite.org/meme/tools/momo). Functional annotation relied on the InterPro domain database (http://www.ebi.ac.uk/interpro/).

### N-glycome analysis

This analysis of H11 was conducted using an established protocol (31). N-glycans were released from glycopeptides by PNGase F. PNGase F-resistant N-glycans were subjected to PNGase A digestion. Following porous graphitic carbon (PGC) purification (32), native glycans were permethylated by adding 100 μL of DMSO-NaOH slurry. Subsequently, the glycan sample was mixed with freshly-prepared 2,5-dihydroxybenzoic acid (DHB) before crystallisation at room temperature. N-glycome spectra were obtained in the positive ionisation mode using an AB 5800 MALDI-TOF-TOF instrument (SCIEX, Concord, Canada). The data were processed using Data Explorer 4.0 (SCIEX) and GlycoWorkbench (v.2.1).

### Treatment of H11 antigen

For experimentation, H11 was used in a native (NA) form; denatured (DN) at 85°C for 25 min (33); periodate (PI)-treated (34), or digested with PNGase F and PNGase A (F+A) (see *N-glycoproteome analysis*) to remove N-glycans (31). Glycan moieties of the H11 antigen were destroyed using an established periodate oxidation procedure (34). Native H11 (1 mg/mL) was incubated in 10 mM sodium periodate (pH 5.0, 45 min at 24°C in the dark) to disrupt glycans without affecting protein (35), and then reduced by 50 mM sodium borohydride for 25 min. Following protein quantitation using the BCA method, all samples were stored at -80°C until use. The H11 samples (NA, DN, PI and F+A) were quantified and concentrations adjusted to 4 μg/mL; the effectiveness of periodate treatment (oxidation) was assessed by direct ELISA (36) and immunoblot (34) using horse radish peroxidase (HRP)-conjugated concanavalin A (EY Laboratories Inc., San Mateo, USA).

### Immunisation trial

To test whether native, denatured- or periodate-treated H11 proteins could induce protective immunity against *H. contortus*, immunisation and infection experiments were conducted in goats (raised helminth-free). All animal experimentation was approved by the Animals Ethics Committee of Huazhong Agricultural University (permit HZAUGO-2019-002). Four-month-old Boer goats, procured from the Hubei Academy of Agricultural Sciences (Wuhan, China), were housed indoors under parasite-free conditions, and provided with fodder twice a day with water *ad libitum*. Four groups were formed with five goats in each group (age- and weight-matched). Goats in group AJ were injected with a saponin-based adjuvant (Quil-A^®^, *In vivo*Gen, San Diego, California, USA; negative control); those in groups NA, DN and PI with native H11 (positive control), denatured H11 or periodate-treated H11 (**Figure 4A**); one animal in group PI died of an unrelated cause on day 56 of the immunisation/challenge experiment. Each goat was immunised subcutaneously thrice at three-week intervals with 150 μg/mL protein equivalent in Quil-A^®^ (37). On the same day after the third immunisation (day 42), all goats were challenged with 7000 infective, third-stage larvae (L3s); on this day, none of the goats excreted nematode eggs in faeces. During this experiment, blood was taken at eight time points (days 0, 10, 21, 31, 42, 49, 63 and 77). Faecal samples were collected rectally from individual goats three times each week, commencing two weeks after the challenge infection (day 56) until the end of the experiment (day 80) (**Figure 4A**). On day 80, goats were euthanised, and adult worms in individual abomasa were enumerated (**Figure 4A**). Tissues were prepared for hematoxylin and eosin (HE) staining and histopathological examination.

### Serum antibody ELISA

Specific anti-H11 IgG, IgM and IgA serum antibody levels (titers) were measured by ELISA (38). In brief, microtitration plates were coated with native H11 (4 μg/mL), blocked with phosphate-buffered saline (PBS; pH 7.4) containing 1% (w/v) bovine serum albumin (PBS-BSA) and washed with the same solution. Individual goat sera were tested at 1/200 dilution, incubated with HRP-conjugated donkey anti-goat IgG (1/500), rabbit anti-goat IgM (1/10,000) or rabbit anti-goat IgA (1/10,000) in PBS-BSA. Plates were developed using tetramethylbenzidine (for 15 min), and plates were read at 630 nm.

### IgG purification and immunoblot

Serum samples from the immunisation trial were examined for IgG antibody responses in the goat groups AJ, NA, DN and PI. First, the serum samples collected from each group on day 42 were pooled (10 mL each). Second, IgG antibodies were purified from each of the four pooled serum samples using Protein A+G agarose (Beyotime Biotechnology, Shanghai, China), according to the manufacturer’s protocol (eluting into 5 mL). Third, these antibody samples were each diluted 1/400 and used to probe immunoblot strips (polyvinylidene fluoride, PVDF) each containing 10 µg of sodium dodecyl sulfate (SDS)-denatured H11, separated in a gradient (4-12%) SDS-PAGE gel (blocked with PBS-BSA) (17); each strip was washed in PBS-BSA and then incubated in HRP-conjugated donkey anti-goat IgG (1/1000), developed for 90 min and scanned on a chemiluminescence imaging system (Tanon 4600, Shanghai, China) and processed using ImageJ software (NIH, USA).

### Passive transfer of IgG antibodies

To evaluate whether IgG antibodies (from goats actively immunised with H11; i.e. group NA) passively transferred to naïve goats (maintained under parasite-free conditions) could achieve protection against *H. contortus* infection, we used the same experimental design as for the immunisation experiment (i.e. assigned four goats to group AJ, and five to each of the groups NA, DN and PI). The amount of IgG antibodies transferred (dose) was as reported in the literature (39). Each goat received IgG antibodies (5 mg) in 50 mL physiological saline intravenously on days 0 and 4, and was infected with 7000 L3s on day 1 of the experiment. Faecal samples were collected six times from day 25 after the challenge to day 35 so that *H. contortus* eggs per gram of faeces could be enumerated. On day 35, all goats were euthanised, and the numbers of worms in individual abomasa were counted (**Figure 6A**).

### Lectin histochemistry and immunohistochemistry

To investigate whether purified IgG antibodies specifically bound native H11 in the gut of *H. contortus*, lectin histochemistry and immunohistochemistry were performed by fluorescent localisation analysis (40). The adult female worms were sliced (4 μm thick), mounted on the slides, then incubated with fluorescein-labelled concanavalin A (Vector Laboratories) and stained with DAPI to detect cell nuclei. For co-localisation, slides were incubated first with fluorescein-labelled concanavalin A and then with IgG antibodies representing the goat-groups AJ, NA, DN or PI, respectively. After extensive washing, the samples were incubated with Cy3-conjugated donkey anti-goat IgG (Beyotime Biotechnology) and then 4’,6-diamidino-2-phenylindole (DAPI). The slides were mounted with an anti-fading solution for fluorescence microscopy (Olympus, Tokyo, Japan).

### IgG antibody inhibition assays

To assess whether IgG antibodies from goats immunised with native or treated H11 could inhibit aminopeptidase activity, the intestinal tracts from 20 fresh, live adult *H. contortus* were collected (41), homogenized in 200 µL of PBS. The homogenate (10 μg protein per well) (42) was mixed with 0.2 mM L-leucine-p-nitroanilide (L-Leu-pNA) at pH 3.0 to 8.0 to assess the optimal pH of aminopeptidase. To investigate the inhibition effect of respective IgG antibodies at an optimal pH, protein samples were pre-incubated with 5 μL of IgG antibodies (1 mg/mL) purified from the normal pre-immunisation sera or sera from goats in groups AJ, NA, DN or PI (see *IgG purification and immunoblot*) for 15 min, and then incubated with L-Leu-pNA substrate for 2.5 h. The inhibitor sensitivity assay (positive control) was carried out by pre-incubation with 10 μM bestatin before adding substrate (17). The absorbance was determined at 405 nm using a multi-mode plate reader (BioTek Cytation 5, Winooski, Vermont, USA).

To assess whether IgG antibodies could inhibit larval development, xL3s were cultured in 24-well plates (100 xL3s/well) in 200 μL of sterile Luria Bertani (LB) medium supplemented with 100 IU/mL of penicillin, 100 µg/mL of streptomycin and 0.25 µg/mL of amphotericin (Antibiotic-Antimycotic, Sigma-Aldrich, St. Louis, Missouri, USA) (43) and exposed to IgG antibodies (1 µg/µL; 50 μL aliquots) from each group (normal, AJ, NA, DN or PI; in triplicate), and incubated at 39°C and 20% CO_2_. The developmental rate was assayed on days 3 and 4 by examining the presence of a buccal capsule, characteristic of the L4 stage (44). The length and width of individual L4s were measured on day 7.

### Statistical analyses

All statistical analyses were conducted using Premier 8.0 software (GraphPad, La Jolla, USA), and standard deviation (SD) or standard error of mean (SEM) calculated. The statistical differences of worm burdens and cumulative FEC were compared by one-way analysis of variance (ANOVA) followed by Dunnett test. The *p < 0.05, **p < 0.01, ***p < 0.001, ****p < 0.0001 and *ns* (nonsignificant) indicate the degree of significance.

## Results

### N-glycoproteome, and glycan profiles and moieties

**Figure 1:**
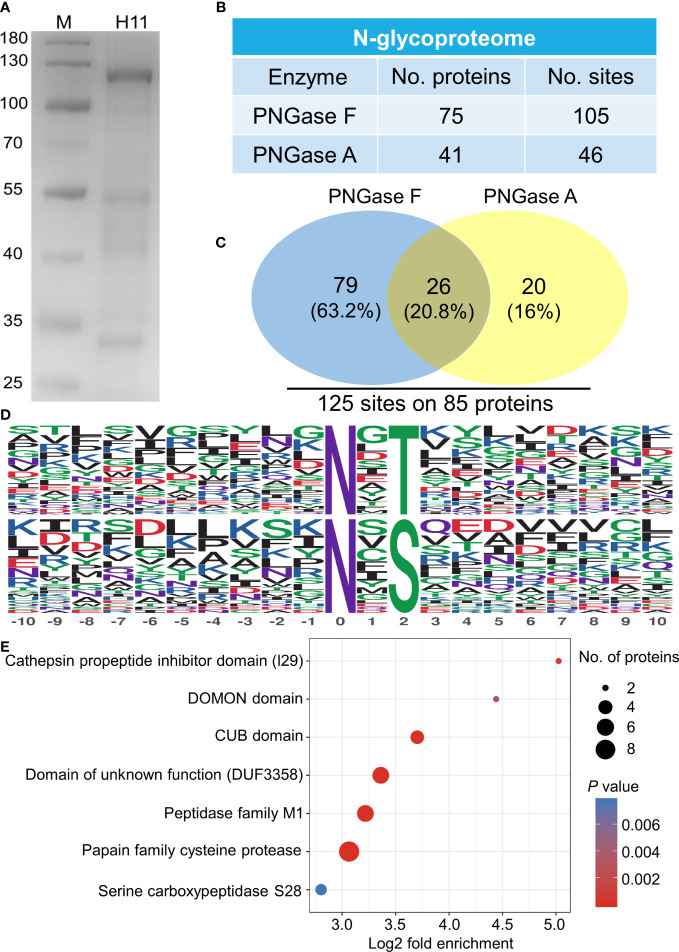
N-glycoproteome analysis of *Haemonchus contortus* H11. **(A)** SDS-PAGE analysis of concanavalin A lectin-purified *H. contortus* native H11 (10 µg). **(B)** N-glycoproteome identified in native H11. H11 sample was sequentially digested with PNGase F and PNGase A, and their N-glycoproteins and N-glycosylation sites were identified by liquid chromatography-tandem mass spectrometry (LC-MS/MS). Data are derived from two independent experiments (cf. **Supplementary Table 1**). **(C)** Comparison between the PNGase F- and PNGase A-released N-glycosylation sites and total numbers of N-glycoproteins and N-glycosylation sites are shown. **(D)** Conserved sequence motifs analyses around glycosylated asparagine residues (−10 to +10; N, asparagine; T, threonine; S, serine). **(E)** Domain enrichment analysis of the identified N-glycoproteins (*p* < 0.05). Each domain is bubble-coded; red and blue represent the enrichment and depletion (Fisher’s exact test *p* value); size reflects the number of glycoproteins (cf. **Supplementary Table 2**).

A glycoproteomic assessment of native H11, corresponding to bands of 100-130, 40-55 and 30-35 kDa in SDS-PAGE analysis (**Figure 1A**), identified 85 distinct proteins with 125 N-glycosylated sites (**Figures 1B, C**; **Supplementary Table 1**). Two conserved motifs N-x-T and N-x-S were associated with glycosylated asparagine residues (−10 to +10), where x represents all amino acids, except proline (**Figure 1D**). Domain enrichment analysis revealed cysteine protease and peptidase M1 aminopeptidase as the two most abundant glycoproteins (**Figure 1E**; **Supplementary Table 2**).

**Figure 2:**
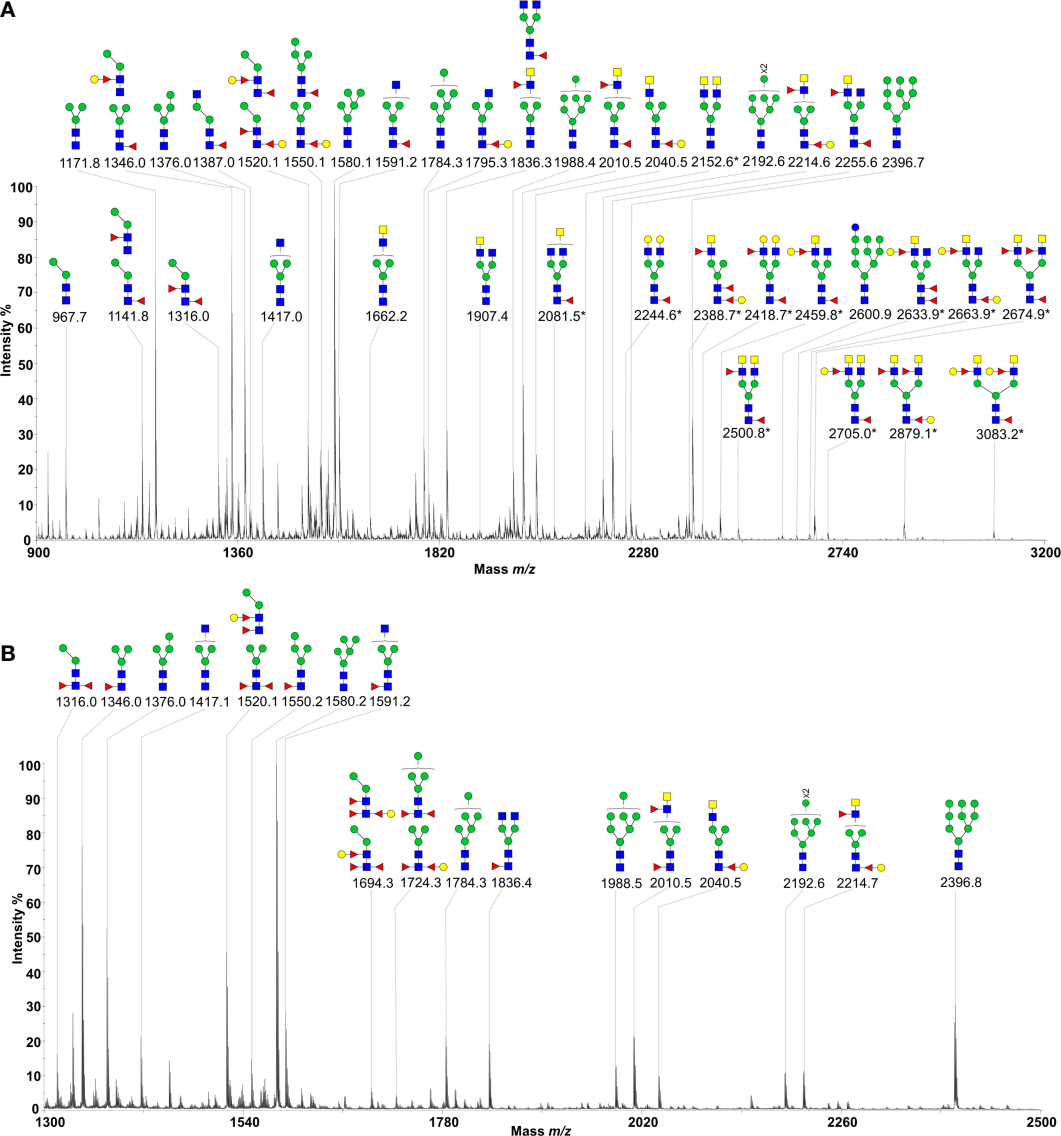
N-glycan profiles of *Haemonchus contortus* H11. H11 N-glycans were fully released by sequential digestion of glycopeptides with PNGase F and PNGase A. Two N-glycan pools were purified, permethylated and then subject to MALDI-TOF-MS analyses. **(A)** MALDI-TOF-MS spectrum of the permethylated N-glycans from *H. contortus* H11 released by PNGase F. The newly-found N-glycan signals are marked with asterisks (cf. **Supplementary Table 3**). **(B)** MALDI-TOF-MS spectrum of the permethylated N-glycans from *H. contortus* H11 released by PNGase A (cf. **Supplementary Table 4**). Glycan species are displayed primarily as [M + Na]^+^ adducts. N-glycan peaks are annotated using the symbol nomenclature (green circle = mannose; yellow circle = galactose; blue circle = glucose; blue square = GlcNAc; yellow square = GalNAc; red triangle = fucose). All N-glycan structures are deduced by the MALDI-TOF-MS/MS fragmentation and the current knowledge of N-glycan biosynthesis in helminths (cf. **Supplementary Tables 3**, **4**).

**Figure 3:**
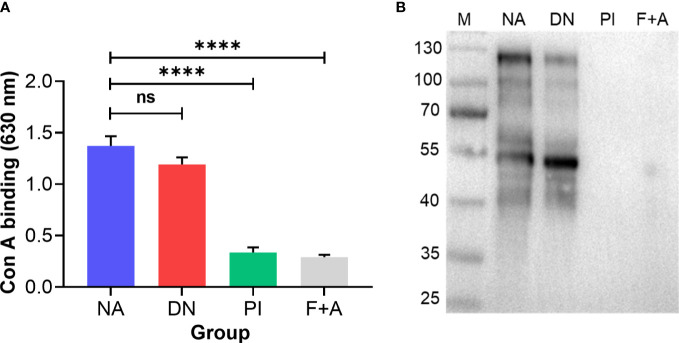
N-glycan moieties on H11 are damaged by periodate treatment. Enzyme-linked immunosorbent assay (ELISA) and immunoblot analysis to assess the N-glycan moieties on H11 samples. Four H11 samples with different treatments including native form (NA), denatured at 85°C for 25 min (DN), periodate treatment (PI), or digested with PNGase F and PNGase A (F+A) to remove N-glycans. **(A)** ELISA of concanavalin A (con A, a lectin that binds to mannose on glycoproteins) binding levels in each H11 sample (NA, DN, PI or F+A) to detect the efficacy of periodate treatment. Data show the mean ± SEM from three independent experiments. Statistical significance was determined by one-way ANOVA and indicated by asterisks, ****p < 0.0001 and ns (not significant). **(B)** Immunoblot of con A binding in each H11 sample (NA, DN, PI or F+A).

MALDI-TOF-MS analysis of H11 identified 38 major signal peaks for permethylated, native glycans. PNGase F-released glycans (*n* = 38) corresponded to the pauci-manosidic structures with or without fucose (Hex_2-4_HexNAc_2_Fuc_0-2_), high-mannose moiety (Hex_5-9_HexNAc_2_) and some complex glycans (Hex_2-5_HexNAc_3-6_Fuc_0-3_) harbouring the major core α1,6 fucose and antenna motifs, including the putative GalNAc-GlcNAc (LDN), fucosylated LDN (LDNF) and/or galactosylated LDNF (**Figure 2A**; **Supplementary Table 3**). PNGase A-released glycans (*n =* 9) represented structures containing core α1,3 fucose residues (**Figure 2B**; **Supplementary Table 4**). The remainder of N-glycans (*n =* 9) had incompletely removed moieties from glycopeptides following PNGase F treatment. Most N-glycan peaks were consistent with those published previously (31), except for novel signals at *m*/*z* 2081.5, 2152.6, 2224.6, 2388.7, 2418.7, 2459.8, 2500.8, 2633.9, 2663.9, 2674.9, 2705.0, 2879.1 and 3083.2, following PNGase F-treatment (**Supplementary Table 3**). Extending this work, we showed that native and denatured H11 bound to concanavalin A (a lectin that binds to mannose on glycoproteins). In contrast, sodium periodate-treated or PNGase (F+A)-treated H11 did not, demonstrating a loss of N-glycan moieties in H11 following each of these treatments (**Figures 3A, B**).

### N-glycan moieties in H11 induce substantial protective immunity and a specific IgG antibody response

**Figure 4:**
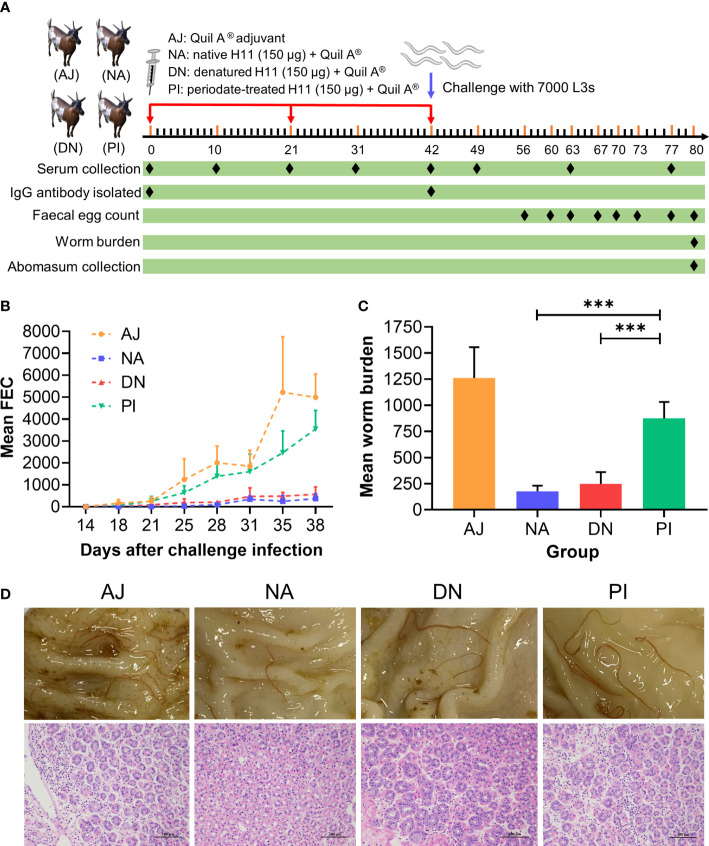
N-glycan moieties on H11 induce prominent protective immunity in goats against *Haemonchus contortus* infection. **(A)** Design of the animal trial. Four groups of five goats each were injected with Quil A^®^ adjuvant alone (AJ), native H11 (NA), denatured H11 (DN) or periodate-treated H11 (PI), respectively, each with 150 μg proteins equivalent in Quil-A^®^. Each goat was immunised subcutaneously three times at three-week intervals (days 0, 21 and 42; red arrow) and then challenged with 7000 infective third-stage larvae (L3s) on day 42 (blue arrow). One goat in group PI died of an unrelated cause. The time points for serum collection (eight times), IgG isolation (twice), faecal sample collection (eight times), worm sample collection (once) and abomasum collection (once) are indicated with black diamond. The numbers of eggs per gram faecal sample (faecal egg count, FEC) were counted under microscopy and normalised to the numbers of adult worms. **(B)** Mean group FEC (mean ± SD; AJ, *n =* 5; NA, *n =* 5; DN, *n =* 5 and PI, *n =* 4) was monitored at eight time points (days 14, 18, 21, 25, 28, 31, 35 and 38 after challenge infection). **(C)** Mean group worm burden (mean ± SD; AJ, *n =* 5; NA, *n =* 5; DN, *n =* 5 and PI, *n =* 4) was counted at the end of the experiment (day 80). Statistical significance was determined using one-way ANOVA, ***p < 0.001. **(D)** Representative images showing the *H. contortus* adults (upper panel) and hematoxylin and eosin(HE) staining (lower panel) in individual abomasa of goats from four groups (AJ, NA, DN and PI). Evident pathological alterations in the abomasum show atrophy and cell necrosis of glands, enhanced eosinophilia and lymphocyte infiltration. Scale bars: 100 µm.

Given that vaccination with native H11 achieves high levels of protection in sheep (45), we assessed whether this protection is linked to protein and/or glycan moieties. To do this, we injected goats subcutaneously three times at three-weekly intervals with either adjuvant, native, denatured or periodate-treated H11 and challenged them (on the same day after the last injection) with 7000 infective third-stage larvae (L3s) of *H. contortus* (**Figure 4A**). Goats immunised separately with native and denatured H11 exhibited a significant decrease (> 93% and 87%, respectively) in the numbers of *H. contortus* eggs in faeces (**Figure 4B**; **Table 1**), and harboured 86% and 80% fewer worms at necropsy on day 38 (following challenge infection) compared with goats that received periodate-treated H11 (37% and 31%, respectively) (**Figure 4C**; **Table 1**); in the latter group of goats, pathological alterations in the abomasa were more pronounced (**Figure 4D**).

**Table 1:** Results from the immunisation trial in goats. Four groups of five goats each were injected with adjuvant alone (AJ), native H11 (NA), denatured H11 (DA) or periodate-treated H11 (PI), respectively and then challenged with 7000 third-stage larvae (L3s) of *H. contortus* (day 42, cf. **Figure 4A**). Faecal samples were taken from individual goats at eight time points (cf. **Figure 4A**), and the number of *H. contortus* eggs per gram was counted (faecal eggcount, FEC). Cumulative FEC of individual goats and mean cumulative FEC (with standard deviations, SD) were calculated for each group. At the end of the experiment (day 80), the numbers of worms in the stomachs (abomasa) were counted, and the reduction in the intensity of infection was calculated for each group.

Group	Faecal egg count (FEC)					Intensity of infection				
	Cumulative FEC	Mean FEC	SD	Reduction^b^ (%)		Number of worms	Mean	SD	Reduction^b^ (%)
AJ	22700	15720.0	5090.7	Not		1355	1260.2	295.3	Not
(*n* = 5)	9000			applicable		939			applicable
	13150					1550			
	13350					954			
	20400					1503			
NA	700	1080.0	555.5	93.1^***^		178	177.0	54.5	86.0^***^
(*n* = 5)	1350					201			
	400					94			
	950					169			
	2000					243			
DN	2200	1990.0	716.5	87.3^***^		282	249.0	111.6	80.2^***^
(*n* = 5)	2650					143			
	1050					216			
	1250					178			
	2800					426			
PI	7450	9950.0	2443.6	36.7^*^		655	874.3	158.1	30.6^ns^
(*n* = 4)^a^	13850					1027			
	8400					881			
	10100					934			

^a^One goat died of cause unrelated to haemonchosis. ^b^For each group, the reduction (%) = 100 – [the mean value for test group ÷ mean value for adjuvant control (AJ) × 100%]. Statistical significance was determined by one -way ANOVA, *p < 0.05, ***p < 0.001, and ns (not significant).

**Figure 5:**
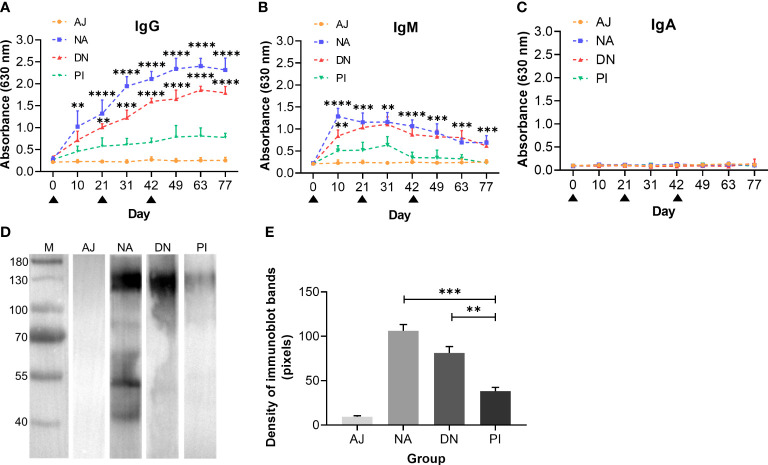
N-glycan moieties on H11 induce a highly-specific anti-H11 IgG antibody response in goats immunised with H11 antigens of *Haemonchus contortus.* Specific anti-H11 subtype antibody (IgG, IgM and IgA) responses in four groups of goats immunised with adjuvant (AJ), native H11 (NA), denatured H11 (DN) and periodate-treated H11 (PI), were determined by enzyme-linked immunosorbent assay (ELISA) and immunoblot analysis (for IgG only). **(A–C)** Dynamics of anti-H11 IgG **(A)**, IgM **(B)** and IgA **(C)** serum antibody responses in four groups of goats (AJ, NA, DN and PI) at eight time points (days 0, 10, 21, 31, 42, 49, 63 and 77, cf. **Figure 4A**). Each data point (absorbance at 630 nm) denoted the mean group antibody titers (mean ± SD; AJ, *n =* 5; NA, *n =* 5; DN, *n =* 5 and PI, *n =* 4). The black triangles indicated three time points of goat immunisation (days 0, 21, and 42, cf. **Figure 4A**) and asterisks represented statistically significant differences compared to Group PI (**p < 0.01, ***p < 0.001 and ****p < 0.0001). **(D)** Immunoblot of IgG antibodies binding to native H11. Four IgG antibodies were purified from pooled sera samples (collected on day 42 of the immunisation trial, cf. **Figure 4A**) from individual animal groups (AJ, NA, DN or PI). **(E)** Densities of four immunoblot bands were quantitatively analysed using the ImageJ program (NIH, USA). Data represent the mean ± SEM from three independent analyses. Statistical significance was determined using one-way ANOVA, **p < 0.01, ***p < 0.001.

We also measured serum antibody levels, and showed that goats immunised with either native or denatured H11 had significantly (*p* < 0.0001) high-specific anti-H11 IgG levels (days 42, 49, 63 and 77) compared with control goats administered either periodate-treated H11 or adjuvant alone (**Figure 5A**). Specific anti-H11 IgM levels were detected on day 10 and decreased gradually from day 31 onward, whereas anti-H11 IgA was not detectable in the sera from any of the four animal groups (**Figures 5B, C**). Immunoblot analysis revealed that bands of 110-130 kDa of high intensity were recognised by IgG serum antibodies from goats immunised with native or denatured H11 compared with controls (**Figures 5D, E**). Overall, these findings provided evidence that the specific IgG antibody response recorded in immunised animals relates to glycan epitopes of H11.

### Passive transfer of protective immunity

**Figure 6:**
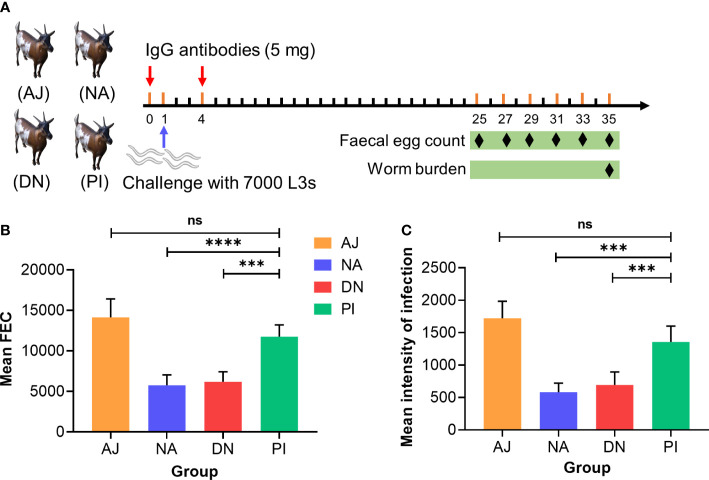
Specific anti-H11 IgG antibodies confer the protection in passive immunisation trials against *Haemonchus contortus* infection. **(A)** Experiment design of passive animal immunisation. Each of four groups of goats (AJ, *n =* 4; NA, *n =* 5; DN, *n =* 5 and PI, *n =* 5) received intravenously twice (days 0 and 4, red arrow) IgG antibodies (5 mg) purified from pooled sera samples (collected on day 42 of the immunisation trial, cf. **Figure 4A**) from individual animal groups, and infected with 7000 infective third-stage larvae (L3s) on day 1 of the experiment (blue arrow). The numbers of eggs per gram faecal sample (faecal egg count, FEC) were microscopically counted at six time points (days 25, 27, 29, 31, 33 and 35, black diamond) and worm burden was counted at the end of the experiment (day 35, black diamond). **(B)** Mean group FEC (mean ± SD; AJ, *n* = 4; NA, *n* = 5; DN, *n* = 5 and PI, *n* = 5) was calculated by the cumulative FEC of each goat. **(C)** Mean group worm burden (mean ± SD; AJ, *n* = 4; NA, *n* = 5; DN, *n* = 5 and PI, *n* = 5) was counted by the numbers of adult worms of each goat. Statistical significance was determined using one-way ANOVA and indicated by asterisks, ***p < 0.001, ****p < 0.0001 and ns (not significant).

Having shown that immunoprotection relates predominantly to glycan moieties, we explored whether a passive transfer of immunity could be achieved. To do this, we transferred IgG antibodies purified from sera from goats with known levels of protection (31-93%) against *H. contortus* (**Figure 4**; **Table 1**) to naïve goats (raised under parasite-free conditions) (**Figure 6A**). We showed that naïve goats that received IgG antibodies from goats immunised with either native or denatured H11 exhibited significantly (*p* ≤ 0.0001) lower *H. contortus* egg numbers in their faeces (**Figure 6B**) and a very (*p* ≤ 0.0006) lower infection intensity (**Figure 6C**) compared with controls receiving IgG antibodies from goats injected with either periodate-treated H11 or adjuvant alone. These results indicated that IgG serum antibodies induced by native H11 antigen could confer passive immunity to naïve goats.

### IgG serum antibodies from immunoprotected goats bind glycoconjugates in the microvilli of the *H. contortus* gut, and inhibit aminopeptidase activity and worm development

**Figure 7:**
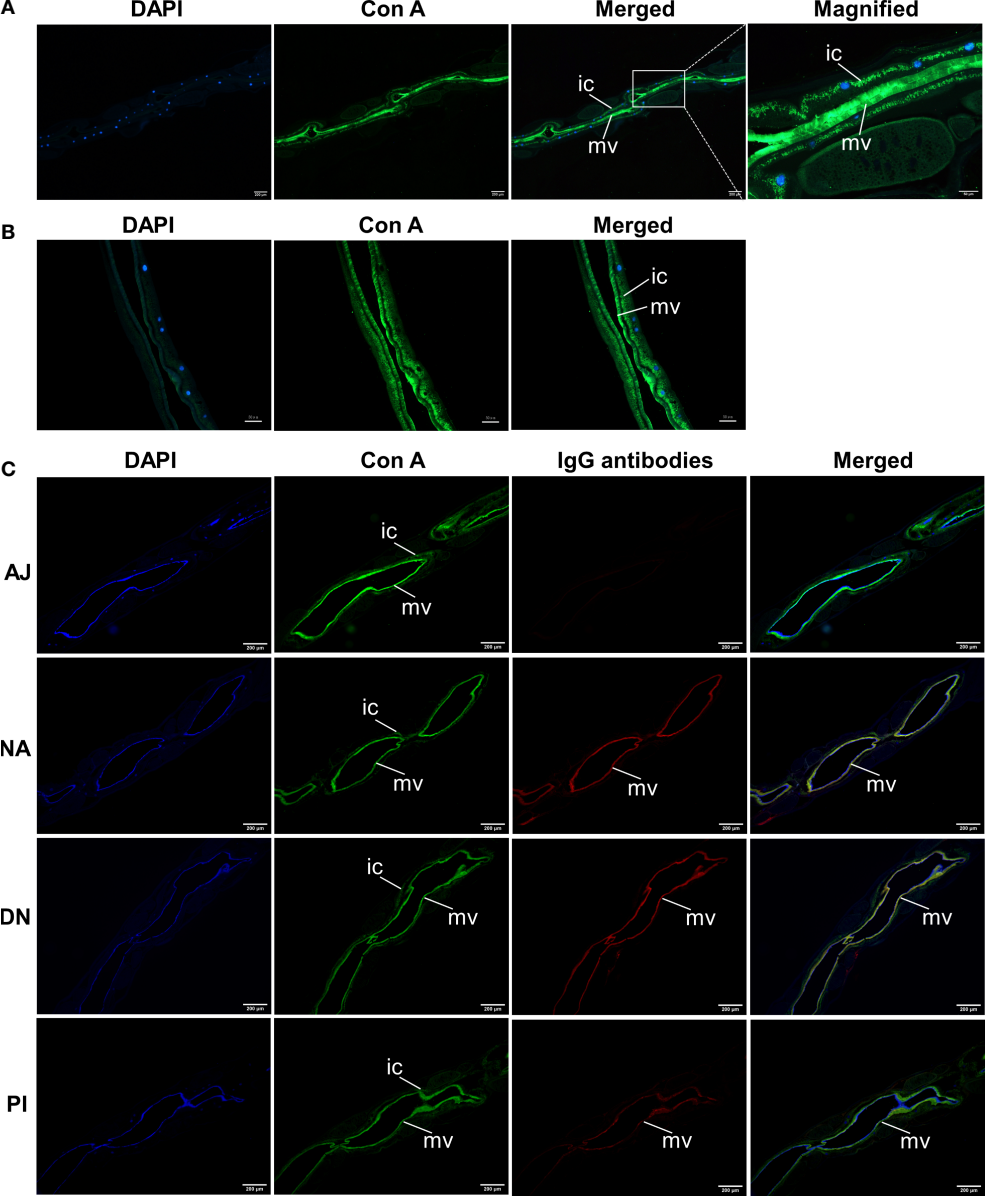
Anti-H11 IgG antibodies from immunoprotected goats bind glycoconjugates in the microvilli of *Haemonchus contortus* gut. **(A, B)** Lectin histochemistry analysis to determine the localisation of native H11 in adult *H. contortus*. Paraffin-embedded slides of adult worm **(A)** and isolated intestinal tissue **(B)** were probed with fluorescein-labelled concanavalin A (Con A, green), and then stained with 4’,6-diamidino-2-phenylindole (DAPI, blue) to detect cell nuclei. Panel 1, DAPI; Panel 2, Con A; Panel 3, merged signals from DAPI and Con A; Panel 4, merged image magnified from panel 3. **(C)** Lectin histochemistry and immunohistochemistry to determine the co-localisation of Con A and IgG antibodies bound native H11 in adult *H. contortus.*Slides of adult worms were incubated with fluorescein-labelled Con A (green) and then with four IgG antibodies purified from pooled sera samples (collected on day 42 of the immunisation trial, cf. **Figure 4A**) from individual animal groups (AJ, NA, DN or PI). Washing samples were incubated with Cy3-conjugated donkey anti-goat IgG (red) and then stained with DAPI (blue). Panel 1, DAPI; Panel 2, Con A; Panel 3, individual IgG antibodies; Panel 4, merged signals from DAPI, Con A and individual IgG antibodies. Scale bars: A = 50 µm or 200 µm; B = 50 µm; C = 200 µm. (ic = intestinal cytoplasm; mv = microvilli). The images shown here were derived from two independent experiments.

**Figure 8:**
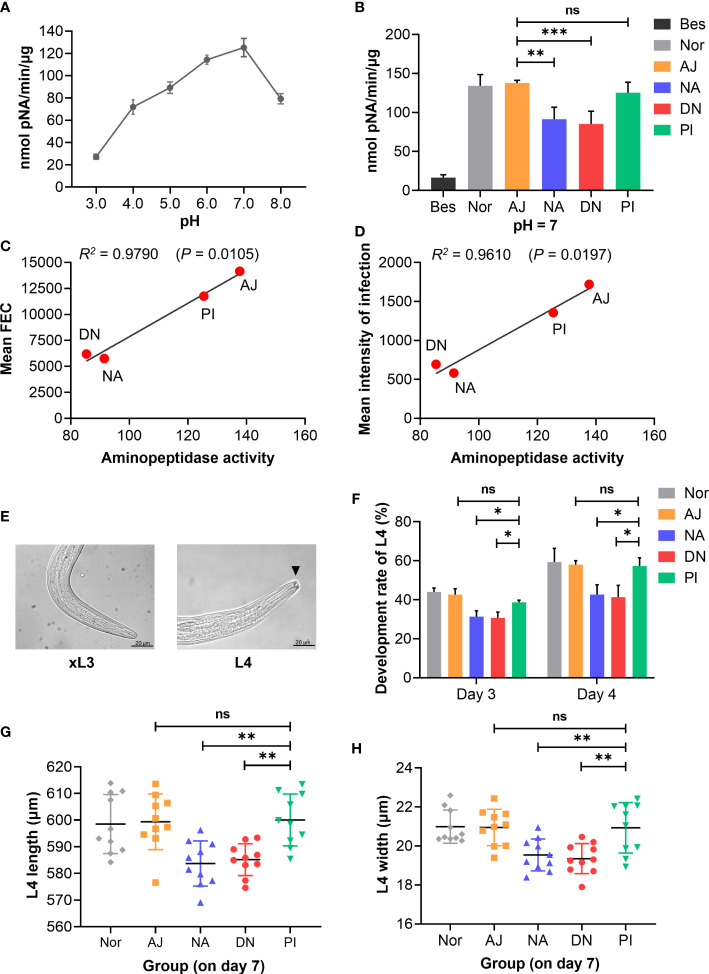
Anti-H11 IgG antibodies from immunoprotected goats inhibit intestinal aminopeptidase activity and *Haemonchus contortus* development. IgG antibody inhibition assays were assessed by inhibiting intestinal aminopeptidase activity and *H. contortus* development *in vitro.* Five IgG antibodies: one was purified from the normal pre-immunisation sera; four were purified from pooled sera samples (collected on day 42 of the immunisation trial, cf. **Figure 4A**) from individual animal groups (AJ, NA, DN or PI). **(A)** Determination of intestinal aminopeptidase activity at pH 3.0 to 8.0 to assess the optimal pH of aminopeptidase. Intestinal homogenate from adult *H. contortus* was incubated with 0.2 mM L-leucine-p-nitroanilide (L-Leu-pNA) substrate. Enzyme activity was detected at 405 nm and expressed as nanomole of pNA/min/µg. **(B)** Inhibition assays of intestinal aminopeptidase activity were performed in the optimal pH by pre-incubation with each group of IgG antibodies (1 µg/µL; 5 μL aliquots) and followed by incubation with L-Leu-pNA substrate. The inhibitor sensitivity assay was carried out by incubation with 10 μM bestatin (Bes). **(C, D)** Correlations of aminopeptidase activity inhibition by each groups of IgG antibodies (AJ, NA, DN or PI) with mean faecal egg count (FEC, **C**) and mean intensity of infection **(D)** in passive immunisation trials. **(E, F)** Exsheathed L3s (xL3s) were cultured in 200 μL of sterile Luria Bertani (LB) medium and then five groups of IgG antibodies (1 µg/µL; 50 μL aliquots) were added to the culture media, respectively. Developmental rate (%) of L4 (*n =* 50) was assessed on day 3 and day 4 **(F)** by examining the presence of a buccal capsule (**E**; arrowhead; Scale bars = 20 µm). **(G, H)** On day 7 of the co-culture with indicated five groups of IgG antibodies, body length **(G)** and width **(H)** of L4 (*n =* 10) were measured (µm). Experiments were independently repeated three times in **(A, B, F, G, H).** Data show as mean ± SEM in **(A, B, F)** or mean ± SD in **(G**, **H)**, and analyse by one-way ANOVA, *p < 0.05, **p < 0.01, ***p < 0.001 and ns (not significant).

Extending previous findings (10, 11), we specifically identified glycoconjugates in the intestine of *H. contortus* adults with fluorescein-labelled concanavalin A. Fluorescence was pronounced in the cytoplasm and microvilli throughout the entire intestine (**Figures 7A, B**). We also showed that IgG antibodies from the serum from goats immunised with native or denatured H11 colocalized with these glycoconjugates, with significantly higher binding intensity compared with antibodies from the periodate-treated and adjuvant controls (**Figure 7C**). For adult worms, we also demonstrated that intestinal aminopeptidase activity could be markedly inhibited by the same IgG antibodies from immunoprotected goats compared with respective controls (**Figures 8A, B**), and that inhibition levels were correlated with the reductions in *H. contortus* egg numbers in faeces and infection intensity (*R^2^* of 0.98 and 0.96 respectively; **Figures 8C, D**).

Subsequently, we assessed whether IgG serum antibodies from immunoprotected goats inhibited the development of *H. contortus in vitro*. We showed that IgG antibodies from goats immunised with native or denatured H11 inhibited the development of exsheathed third-stage larvae (xL3s) to fourth-stage larvae (L4s) (**Figure 8E**), compared with IgG antibodies from control goats either immunised with periodate-treated H11 or injected with adjuvant alone (**Figure 8F**). L4s incubated with IgG antibodies from immunoprotected goats were significantly smaller than those incubated with antibodies from these respective control animals (**Figures 8G, H**).

## Discussion

H11 is the best-studied immunogen (vaccine molecule) from a parasitic nematode (12, 46). In its native form, H11 achieves high levels (usually 75-95%) of protection in animals (8). In the late 1980s, the advent of recombinant DNA technology stimulated researchers in the UK and Australia to focus on developing a well-defined recombinant, subunit vaccine representing H11, in order to be prepared to meet the supply, quality and demand for a commercial vaccine. Despite major efforts in academic-industry collaborations, the select H11 proteins expressed in prokaryotic and eukaryotic expression systems (14, 16, 17) did not achieve significant immuno-protection in animals (13, 47, 48). After more than three decades of research on H11, this result raised pertinent questions about precisely which component(s) of native H11 confer(s) reproducible immunoprotection.

Here, we explored whether particular post-translation modifications are responsible for the immuno-protection induced by H11. Using advanced experimental and molecular tools, we elucidated the N-glycoproteome and N-glycome of H11, and demonstrated that N-glycans are a predominant component that induces protection and an associated IgG serum antibody response in immunised animals (i.e. goats), and that this antibody response leads to a marked inhibition of aminopeptidase activity in the nematode’s intestine as well as worm development. We also showed that the H11 glycoproteome contains at least 85 proteins with 125 N-glycosylated sites, contrasting prior work identifying 23 proteins (49). The high analytical sensitivity of the methodology (including HILIC enrichment) employed here allowed us to discover at least six high-confidence aminopeptidases with 13 N-glycosylation sites relating proteins of 100-130 kDa, some of which are consistent with isoforms found previously (14, 50) (cf. **Supplementary Table 5**). The aminopeptidases represent type II integral membrane proteins with aminopeptidase A and M activities, which are located in the intestinal microvilli of *H. contortus* (10). Aminopeptidase activities have been reported in the blood-feeding parasites, indicating an association with the degradation of haemoglobin peptides into free amino acids (51). Notably, our findings revealed that multiple N-glycosylation sites in aminopeptidases have α1,3-fucose in asparagine-linked GlcNAc residues, known to be highly antigenic in other helminth-host systems (52, 53).

Advanced MALDI-TOF-MS analysis, combined with PNGase F/A digestion of N-glycans, allowed us to infer at least 38 N-glycan structures within H11, which compares with N-glycans in previous studies. The detection of 13 additional high-mass glycans relates to the use of concanavalin A for enrichment before analysis (31, 54, 55). The MS/MS spectra of *m/z* 2674.9, 2879.1 and 3083.2 inferred the presence of two LDNF antennae and two galactosylated LDNF antennae (56) (**Supplementary Figures 1A–C**), which have been reported previously to be associated with anti-parasite responses (57). Interestingly, the proportion (67%) of fucose-containing glycans in the H11 N-glycome is high, harbouring α1,3- and/or α1,6-linked monofucosylated, difucosylated, and uncommon trifucosylated glycan structures. The anti-glycan antibody response induced by recombinant *C. elegans* H11, but the α1,3- and/or α1,6-linked difucosylated and trifucosylated N-glycans did not achieve a protective effect (17). This may be explained by the distinctiveness in the N-glycans compared with native H11. Alternatively, additional glycan moieties present in native H11-enriched extract (compared with recombinant *C. elegans* H11) may induce a protective antibody response. In immunobiological studies of the blood fluke *Schistosoma mansoni* (class Trematoda), core α1,3- and α1,6-fucose di-fucosylation and LDNFs have been detected on the egg-derived antigen omega-1 (58). This fucose-linked epitope is reported to play a critical role in stimulating a Th2 phenotype by mediating the internalisation of omega-1 *via* a mannose receptor on dendritic cells (59). Similar, immunologically relevant functions of glycan structures have been inferred for other parasitic helminths, such as *Fasciola hepatica* (liver fluke – a trematode) and *Dictyocaulus viviparus* (lungworm – a nematode) (60, 61).

The present immunisation experiments in goats demonstrated that the de-glycosylation of H11 by periodate treatment significantly impaired immunoprotection and reduced specific anti-H11 IgG serum antibody levels. We infer that glycan moieties present in the glycoproteins of intestinal microvilli induce a particular IgG response that, in turn, targets and destroys the integrity of the nematode’s intestine in the immunised animal, and we demonstrate that IgG antibodies target glycans in the intestine and inhibit the aminopeptidase activity, which correlates with immunoprotection. Importantly, we provide the first experimental evidence that N-glycosylation of the H11 glycoprotein complex in the intestinal microvilli of *H. contortus* is crucial for immunoprotection. Taken together, these findings provide valuable insights and a solid foundation for the future development of vaccines against parasitic nematodes more broadly.

## Data availability statement

The datasets presented in this study can be found in online repositories. The name of the repository and accession number can be found below: EMBL-EBI PRIDE Archive; PXD037140.

## Ethics statement

This study was reviewed and approved by the Animals Ethics Committee of Huazhong Agricultural University (permit HZAUGO-2019-002).

## Author contributions

MH conceived and designed the project with the input from CW, NG, and RBG. CW performed the experiments and analyzed data. LL and TW assisted with animal experiments. XL contributed to N-glycan analysis. WP, RKS, and X-QZ contributed materials. CW, MH, and RBG wrote the manuscript, with inputs from X-QZ and NG. All authors contributed to the article and approved the submitted version.

## Funding

This research project was supported by the National Natural Science Foundation of China (NSFC; grant nos. 31872462 and 32172881) to MH. RBG’s research was supported predominantly by grants from the Australian Research Council (ARC).

## Conflict of interest

The authors declare that the research was conducted in the absence of any commercial or financial relationships that could be construed as a potential conflict of interest.

## Publisher’s note

All claims expressed in this article are solely those of the authors and do not necessarily represent those of their affiliated organizations, or those of the publisher, the editors and the reviewers. Any product that may be evaluated in this article, or claim that may be made by its manufacturer, is not guaranteed or endorsed by the publisher.

## Supplementary material

Supplementary Figure 1 MALDI-TOF-MS/MS spectra of the permethylated N-glycans at *m/z* 2675, 2879 and 3083. **(A–C)** Permethylated N-glycans at *m/z* 2675 **(A)**, 2879 **(B)** and 3083 **(C)** MALDI-TOF-MS/MS analyses: the predicted N-glycan configurations are symbol nomenclature: green circle = mannose; yellow circle = galactose; blue square = GlcNAc; yellow square = GalNAc; red triangle = fucose. Peaks indicated by red arrows with doted lines represent the loss of indicated fragments from the N-glycan ion.

Supplementary Table 1 List of N-glycoproteins and N-glycosylation sites identified in native H11 from *Haemonchus contortus.*

Supplementary Table 2 Results from domain enrichment analysis of N-glycoproteins of native H11 from *Haemonchus contortus.*

Supplementary Table 3 N-glycan configurations predicted for native H11 from *Haemonchus contortus* released by PNGase F.

Supplementary Table 4 N-glycan configurations predicted for native H11 from *Haemonchus contortus* released by PNGase A.

Supplementary Table 5 List of aminopeptidases and their N-glycosylation sites identified in native H11 from *Haemonchus contortus.*

## References

[1] Hodda M. Phylum nematoda: A classification, catalogue and index of valid genera, with a census of valid species. Zootaxa (2022) 5114(1):1–289. doi: 10.11646/zootaxa.5114.1.1 35391386

[2] Hotez PJ Fenwick A Savioli L Molyneux DH. Rescuing the bottom billion through control of neglected tropical diseases. Lancet (2009) 373(9674):1570–5. doi: 10.1016/s0140-6736(09)60233-6 19410718

[3] Jourdan PM Lamberton PHL Fenwick A Addiss DG. Soil-transmitted helminth infections. Lancet (2018) 391(10117):252–65. doi: 10.1016/s0140-6736(17)31930-x 28882382

[4] Brown TL Airs PM Porter S Caplat P Morgan ER. Understanding the role of wild ruminants in anthelmintic resistance in livestock. Biol Lett (2022) 18(5):20220057. doi: 10.1098/rsbl.2022.0057 35506237 PMC9065971

[5] Kaplan RM Vidyashankar AN. An inconvenient truth: global worming and anthelmintic resistance. Vet Parasitol (2012) 186(1-2):70–8. doi: 10.1016/j.vetpar.2011.11.048 22154968

[6] Bethony JM Loukas A Hotez PJ Knox DP. Vaccines against blood-feeding nematodes of humans and livestock. Parasitology (2006) 133(2):63–79. doi: 10.1017/s0031182006001818 17274849

[7] Hotez PJ Diemert D Bacon KM Beaumier C Bethony JM Bottazzi ME. The human hookworm vaccine. Vaccine (2013) 31(2):227–32. doi: 10.1016/j.vaccine.2012.11.034 PMC3988917 23598487

[8] Smith TS Munn EA Graham M Tavernor AS Greenwood CA. Purification and evaluation of the integral membrane protein H11 as a protective antigen against. Haemonchus contortus. Int J Parasitol (1993) 23(2):271–80. doi: 10.1016/0020-7519(93)90150-w 8496010

[9] Smith WD Smith SK Murray JM. Protection studies with integral membrane fractions of. Haemonchus contortus. Parasite Immunol (1994) 16(5):231–41. doi: 10.1111/j.1365-3024.1994.tb00345.x 8072767

[10] Knox DP Redmond DL Newlands GF Skuce PJ Pettit D Smith WD. The nature and prospects for gut membrane proteins as vaccine candidates for *Haemonchus contortus* and other ruminant trichostrongyloids. Int J Parasitol (2003) 33(11):1129–37. doi: 10.1016/s0020-7519(03)00167-x 13678629

[11] Newton SE Munn EA. The development of vaccines against gastrointestinal nematode parasites, particularly Haemonchus contortus. Parasitol Today (1999) 15(3):116–22. doi: 10.1016/s0169-4758(99)01399-x 10322325

[12] Britton C Emery DL McNeilly TN Nisbet AJ Stear MJ. The potential for vaccines against scour worms of small ruminants. Int J Parasitol (2020) 50(8):533–53. doi: 10.1016/j.ijpara.2020.04.003 32569640

[13] Nisbet AJ Meeusen EN Gonzalez JF Piedrafita DM. Immunity to *Haemonchus contortus* and vaccine development. Adv Parasitol (2016) 93:353–96. doi: 10.1016/bs.apar.2016.02.011 27238008

[14] Newton SE Meeusen EN. Progress and new technologies for developing vaccines against gastrointestinal nematode parasites of sheep. Parasite Immunol (2003) 25(5):283–96. doi: 10.1046/j.1365-3024.2003.00631.x 12969446

[15] Yan R Li X. Expression of recombinant H11 of *Haemonchus contortus* in *Pichia pastoris* . J Nanjing Agricult Univ (2005) 28(2):85–9.

[16] Reszka N Rijsewijk FA Zelnik V Moskwa B Bienkowska-Szewczyk K. *Haemonchus contortus*: Characterization of the baculovirus expressed form of aminopeptidase H11. Exp Parasitol (2007) 117(2):208–13. doi: 10.1016/j.exppara.2007.03.018 17482594

[17] Roberts B Antonopoulos A Haslam SM Dicker AJ McNeilly TN Johnston SL. Novel expression of *Haemonchus contortus* vaccine candidate aminopeptidase H11 using the free-living nematode *Caenorhabditis elegans* . Vet Res (2013) 44(1):111. doi: 10.1186/1297-9716-44-111 24289031 PMC4176091

[18] Zhou Q Yang Y Guo X Duan L Chen X Yan B. Expression of *Caenorhabditis elegans*-expressed Trans-HPS, partial aminopeptidase H11 from *Haemonchus contortus* . Exp Parasitol (2014) 145:87–98. doi: 10.1016/j.exppara.2014.08.005 25128369

[19] Laing R Kikuchi T Martinelli A Tsai IJ Beech RN Redman E. The genome and transcriptome of *Haemonchus contortus*, a key model parasite for drug and vaccine discovery. Genome Biol (2013) 14(8):R88. doi: 10.1186/gb-2013-14-8-r88 23985316 PMC4054779

[20] Kooyman FN Ploeger HW Höglund J VAN Putten JPM. Differential N-glycan- and protein-directed immune responses in *Dictyocaulus viviparus*-infected and vaccinated calves. Parasitology (2007) 134(2):269–79. doi: 10.1017/s0031182006001405 17032477

[21] Hokke CH van Diepen A. Helminth glycomics N-glycan repertoires and host-parasite interactions. Mol Biochem Parasitol (2017) 215:47–57. doi: 10.1016/j.molbiopara.2016.12.001 27939587

[22] Lai JD Swystun LL Cartier D Nesbitt K Zhang C Hough C. N-linked glycosylation modulates the immunogenicity of recombinant human factor VIII in hemophilia a mice. Haematologica (2018) 103(11):1925–36. doi: 10.3324/haematol.2018.188219 PMC6278987 30002126

[23] Srivastava AD Unione L Bunyatov M Gagarinov IA Delgado S Abrescia NGA. Chemoenzymatic synthesis of complex N-glycans of the parasite *S. mansoni* to examine the importance of epitope presentation on DC-SIGN recognition. Angew Chem Int Ed Engl (2021) 60(35):19287–96. doi: 10.1002/anie.202105647 PMC8456914 34124805

[24] Cipollo JF Parsons LM. Glycomics and glycoproteomics of viruses: Mass spectrometry applications and insights toward structure-function relationships. Mass Spectrom Rev (2020) 39(4):371–409. doi: 10.1002/mas.21629 32350911 PMC7318305

[25] North SJ Hitchen PG Haslam SM Dell A. Mass spectrometry in the analysis of N-linked and O-linked glycans. Curr Opin Struct Biol (2009) 19(5):498–506. doi: 10.1016/j.sbi.2009.05.005 19577919 PMC2965404

[26] Ruhaak LR Xu G Li Q Goonatilleke E Lebrilla CB. Mass spectrometry approaches to glycomic and glycoproteomic analyses. Chem Rev (2018) 118(17):7886–930. doi: 10.1021/acs.chemrev.7b00732 PMC7757723 29553244

[27] West CM Malzl D Hykollari A Wilson IBH. Glycomics, glycoproteomics, and glycogenomics: An inter-taxa evolutionary perspective. Mol Cell Proteomics (2021) 20:100024. doi: 10.1074/mcp.R120.002263 32994314 PMC8724618

[28] Schwarz EM Korhonen PK Campbell BE Young ND Jex AR Jabbar A. The genome and developmental transcriptome of the strongylid nematode *Haemonchus contortus* . Genome Biol (2013) 14(8):R89. doi: 10.1186/gb-2013-14-8-r89 23985341 PMC4053716

[29] Preston S Jabbar A Nowell C Joachim A Ruttkowski B Baell J. Low cost whole-organism screening of compounds for anthelmintic activity. Int J Parasitol (2015) 45:333–43. doi: 10.1016/j.ijpara.2015.01.007 25746136

[30] Liu X Ma L Li J. Recent developments in the enrichment of glycopeptides for glycoproteomics. Anal Lett (2008) 41(2):268–77. doi: 10.1080/00032710701792711

[31] Wang C Gao W Yan S Zhu X-Q Suo X Liu X. N-glycome and N-glycoproteome of a hematophagous parasitic nematode *Haemonchus* . Comput Struct Biotechnol J (2021) 19:2486–96. doi: 10.1016/j.csbj.2021.04.038 PMC8113779 34025939

[32] Liu X Zhang G Chan K Li J. Microwave-assisted kochetkov amination followed by permanent charge derivatization: A facile strategy for glycomics. Chem Commun (2010) 46(39):7424–6. doi: 10.1039/c0cc01732a 20830339

[33] Klaver EJ Kuijk LM Laan LC Kringel H van Vliet SJ Bouma G. *Trichuris suis*-induced modulation of human dendritic cell function is glycan-mediated. Int J Parasitol (2013) 43(3-4):191–200. doi: 10.1016/j.ijpara.2012.10.021 23220043

[34] Gong W Huang F Ma Y Bai H Yin L Li J. Protective immunity against *Schistosoma japonicum* infection can be provided by IgG antibodies towards periodate-sensitive or periodate-resistant glycans. Parasit Vectors (2015) 8:234. doi: 10.1186/s13071-015-0842-1 25907161 PMC4408597

[35] Velupillai P Secor WE Horauf AM Harn DA. B-1 cell (CD5+B220+) outgrowth in murine schistosomiasis is genetically restricted and is largely due to activation by polylactosamine sugars. J Immunol (1997) 158(1):338–44. 8977208

[36] Lee HH Wang YN Xia W Chen CH Rau KM Ye L. Removal of n-linked glycosylation enhances PD-L1 detection and predicts anti-PD-1/PD-L1 therapeutic efficacy. Cancer Cell (2019) 36(2):168–78. doi: 10.1016/j.ccell.2019.06.008 PMC6793936 31327656

[37] Meier L Torgerson PR Hertzberg H. Vaccination of goats against *Haemonchus contortus* with the gut membrane proteins H11/H-gal-GP. Vet Parasitol (2016) 229:15–21. doi: 10.1016/j.vetpar.2016.08.024 27809971

[38] Lu M Tian X Zhang Y Wang W Tian AL Aimulajiang K. Protection studies of an excretory-secretory protein HcABHD against *Haemonchus contortus* infection. Vet Res (2021) 52(1):3. doi: 10.1186/s13567-020-00871-0 33407892 PMC7786147

[39] Tian X Lu M Jia C Bu Y Aimulajiang K Zhang Y. *Haemonchus contortus* transthyretin domain - containing protein (HcTTR): A promising vaccine candidate against *Haemonchus contortus* infection. Vet Parasitol (2020) 279:109045. doi: 10.1016/j.vetpar.2020.109045 32045836

[40] Fauquenoy S Morelle W Hovasse A Bednarczyk A Slomianny C Schaeffer C. Proteomics and glycomics analyses of N-glycosylated structures involved in *Toxoplasma gondii*-host cell interactions. Mol Cell Proteomics (2008) 7(5):891–910. doi: 10.1074/mcp.M700391-MCP200 18187410

[41] Munn EA Greenwood CA. Endotube-brush border complexes dissected from the intestines of *Haemonchus contortus* and *Ancylostoma caninum* . Parasitology (1983) 87(1):129–37. doi: 10.1017/s0031182000052471 6684761

[42] Shompole S Jasmer DP. Cathepsin B-like cysteine proteases confer intestinal cysteine protease activity in *Haemonchus contortus* . J Biol Chem (2001) 276(4):2928–34. doi: 10.1074/jbc.M007321200 11032834

[43] Ma G Wang T Korhonen PK Young ND Nie S Ang CS. Dafachronic acid promotes larval development in *Haemonchus contortus* by modulating dauer signalling and lipid metabolism. PloS Pathog (2019) 15(7):e1007960. doi: 10.1371/journal.ppat.1007960 31335899 PMC6677322

[44] Sommerville RI. The development of *Haemonchus contortus* to the fourth stage *in vitro* . J Parasitol (1966) 52(1):127–36. doi: 10.2307/3276403 5910446

[45] Munn EA Smith TS Smith H James FM Smith FC Andrews SJ. Vaccination against *Haemonchus contortus* with denatured forms of the protective antigen H11. Parasite Immunol (1997) 19(6):243–8. doi: 10.1046/j.1365-3024.1997.d01-205.x 9364553

[46] Dalton JP Mulcahy G. Parasite vaccines - a reality? Vet Parasitol (2001) 98(1-3):149–67. doi: 10.1016/s0304-4017(01)00430-7 11516584

[47] Matthews JB Geldhof P Tzelos T Claerebout E. Progress in the development of subunit vaccines for gastrointestinal nematodes of ruminants. Parasite Immunol (2016) 38(12):744–53. doi: 10.1111/pim.12391 27726158

[48] Wang C Li F Zhang Z Yang X Ahmad AA Li X. Recent research progress in China on *Haemonchus contortus* . Front Microbiol (2017) 8:1509. doi: 10.3389/fmicb.2017.01509 28883809 PMC5574212

[49] Dicker AJ Inglis NF Manson ED Subhadra S Illangopathy M Muthusamy R. Proteomic analysis of *Mecistocirrus digitatus* and *Haemonchus contortus* intestinal protein extracts and subsequent efficacy testing in a vaccine trial. PloS Negl Trop Dis (2014) 8(6):e2909. doi: 10.1371/journal.pntd.0002909 24901227 PMC4046941

[50] Mohandas N Young ND Jabbar A Korhonen PK Koehler AV Hall RS. The complement of family M1 aminopeptidases of *Haemonchus contortus* - biotechnological implications. Biotechnol Adv (2016) 34(2):65–76. doi: 10.1016/j.biotechadv.2015.10.003 26597954

[51] Williamson AL Brindley PJ Knox DP Hotez PJ Loukas A. Digestive proteases of blood-feeding nematodes. Trends Parasitol (2003) 19(9):417–23. doi: 10.1016/s1471-4922(03)00189-2 12957519

[52] Prasanphanich NS Mickum ML Heimburg-Molinaro J Cummings RD. Glycoconjugates in host-helminth interactions. Front Immunol (2013) 4:240. doi: 10.3389/fimmu.2013.00240 24009607 PMC3755266

[53] Veríssimo CM Graeff-Teixeira C Jones MK Morassutti AL. Glycans in the roles of parasitological diagnosis and host-parasite interplay. Parasitology (2019) 146(10):1217–32. doi: 10.1017/s0031182019000465 31057132

[54] Haslam SM Coles GC Munn EA Smith TS Smith HF Morris HR. *Haemonchus contortus* glycoproteins contain N-linked oligosaccharides with novel highly fucosylated core structures. J Biol Chem (1996) 271(48):30561–70. doi: 10.1074/jbc.271.48.30561 8940027

[55] Paschinger K Wilson IB. Two types of galactosylated fucose motifs are present on N-glycans of *Haemonchus contortus* . Glycobiology (2015) 25(6):585–90. doi: 10.1093/glycob/cwv015 PMC4414472 25740940

[56] Smit CH van Diepen A Nguyen DL Wuhrer M Hoffmann KF Deelder AM. Glycomic analysis of life stages of the human parasite *Schistosoma mansoni* reveals developmental expression profiles of functional and antigenic glycan motifs. Mol Cell Proteomics (2015) 14(7):1750–69. doi: 10.1074/mcp.M115.048280 PMC4587318 25883177

[57] Prasanphanich NS Luyai AE Song X Heimburg-Molinaro J Mandalasi M Mickum M. Immunization with recombinantly expressed glycan antigens from *Schistosoma mansoni* induces glycan-specific antibodies against the parasite. Glycobiology (2014) 24(7):619–37. doi: 10.1093/glycob/cwu027 PMC4038251 24727440

[58] Meevissen MH Wuhrer M Doenhoff MJ Schramm G Haas H Deelder AM. Structural characterization of glycans on omega-1, a major *Schistosoma mansoni* egg glycoprotein that drives Th2 responses. J Proteome Res (2010) 9(5):2630–42. doi: 10.1021/pr100081c 20178377

[59] Everts B Hussaarts L Driessen NN Meevissen MH Schramm G van der Ham AJ. Schistosome-derived omega-1 drives Th2 polarization by suppressing protein synthesis following internalization by the mannose receptor. J Exp Med (2012) 209(10):1753–67. doi: 10.1084/jem.20111381 PMC3457738 22966004

[60] van Die I Cummings RD. Glycan gimmickry by parasitic helminths: A strategy for modulating the host immune response? Glycobiology (2010) 20(1):2–12. doi: 10.1093/glycob/cwp140 19748975

[61] van Die I Cummings RD. The mannose receptor in regulation of helminth-mediated host immunity. Front Immunol (2017) 8:1677. doi: 10.3389/fimmu.2017.01677 29238348 PMC5712593

