# Supplementary figures and images for: H11-induced immunoprotection is predominantly linked to N-glycan moieties during Haemonchus contortus infection
Source: Front Immunol. 2022 Oct 25;13:1034820. doi: 10.3389/fimmu.2022.1034820 (PMC9667387; doi:10.3389/fimmu.2022.1034820)

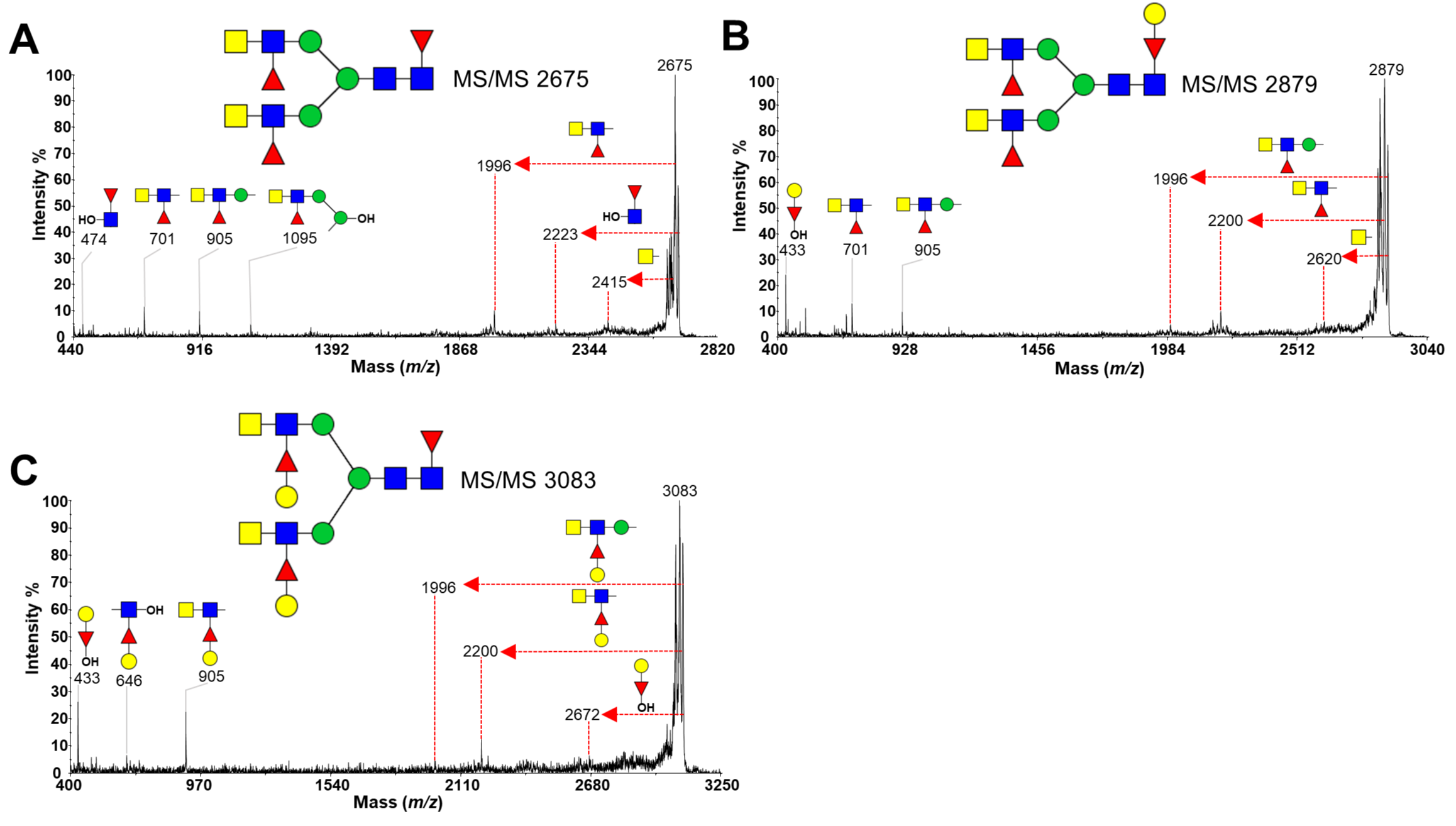

Supplement: Supplementary Figure 1 — MALDI-TOF-MS/MS spectra of the permethylated N-glycans at m/z 2675, 2879 and 3083. (A–C) Permethylated N-glycans at m/z 2675 (A), 2879 (B) and 3083 (C) MALDI-TOF-MS/MS analyses: the predicted N-glycan configurations are symbol nomenclature: green circle = mannose; yellow circle = galactose; blue square = GlcNAc; yellow square = GalNAc; red triangle = fucose. Peaks indicated by red arrows with doted lines represent the loss of indicated fragments from the N-glycan ion. [file Image_1.tif]
