# Supplementary material for: H11-induced immunoprotection is predominantly linked to N-glycan moieties during Haemonchus contortus infection
Source: Front Immunol. 2022 Oct 25;13:1034820. doi: 10.3389/fimmu.2022.1034820 (PMC9667387; doi:10.3389/fimmu.2022.1034820)
Supplement: Supplementary Table 2 — Results from domain enrichment analysis of N-glycoproteins of native H11 from Haemonchus contortus. [file Table_2.docx]

**SUPPLEMENTARY TABLE 2 |** Results from domain enrichment analysis of N-glycoproteins of native H11 from *Haemonchus contortus.*

| **Domain description** | **Mapping** | **Background** | **Mapping**  **(all)** | **Background (all)** | **Fold enrichment** | **Fisher's exact test (*p* value)** | **-Log10 (*p* value)** | **Protein accession nos.** |
| --- | --- | --- | --- | --- | --- | --- | --- | --- |
| Papain family cysteine protease | 9 | 35 | 41 | 1335 | 8.37 | 3.86E-07 | 6.41 | Q8MU53; Q8MM13; Q25030; Q58HK5; C6KI85; Q25032; J9SQ04; F8UA90; A0A2P1E0E6 |
| Domain of unknown function (DUF3358) | 6 | 19 | 41 | 1335 | 10.28 | 1.17E-05 | 4.93 | Q967C6; V5K5H8; A0A126UB22; Q9U5P5; A0A126UAR8; A0A140EQK0 |
| Peptidase family M1 | 6 | 21 | 41 | 1335 | 9.3 | 2.23E-05 | 4.65 | Q967C6; V5K5H8; A0A126UB22; Q9U5P5; A0A126UAR8; A0A140EQK0 |
| CUB domain | 4 | 10 | 41 | 1335 | 13.02 | 0.000141 | 3.85 | W6NST2; W6NE70; W6NDD9; D5FM34 |
| Cathepsin propeptide inhibitor domain (I29) | 2 | 2 | 41 | 1335 | 32.56 | 0.000921 | 3.04 | Q8MU53; Q8MM13 |
| DOMON domain | 2 | 3 | 41 | 1335 | 21.71 | 0.002709 | 2.57 | W6NS28; W6NS31 |
| Serine carboxypeptidase S28 | 3 | 14 | 41 | 1335 | 6.98 | 0.007739 | 2.11 | A5CG76; A5CG77; W6NFT9 |
