# Supplementary material for: H11-induced immunoprotection is predominantly linked to N-glycan moieties during Haemonchus contortus infection
Source: Front Immunol. 2022 Oct 25;13:1034820. doi: 10.3389/fimmu.2022.1034820 (PMC9667387; doi:10.3389/fimmu.2022.1034820)
Supplement: Supplementary Table 3 — N-glycan configurations predicted for native H11 from Haemonchus contortus released by PNGase F. [file Table_3.docx]

**SUPPLEMENTARY TABLE 3 |** N-glycan configurations predicted for native H11 from *Haemonchus contortus* released by PNGase F.

| **No.** | **Observed *m/z***  **[M + Na]^+^** | **Calculated *m/z* [M + Na]^+^** | **Composition** | **Relative**  **abundance (%)^a^** | **Proposed structures** | **Key MS/MS fragments**  ***m/z*** |
| --- | --- | --- | --- | --- | --- | --- |
| 1 | 967.72 | 967.48 | Hex_2_HexNAc_2_ | 2.60 |  |  |
| 2 | 1141.84 | 1141.5 | Hex_2_HexNAc_2_Fuc_1_ | 3.69 |   | B-ions: 690, 864  Y-ion: 474  Z-ion: 935 |
| 3 | 1171.85 | 1171.58 | Hex_3_HexNAc_2_ | 8.80 |  |  |
| 4 | 1315.98 | 1315.66 | Hex_2_HexNAc_2_Fuc_2_ | 2.30 |  | B-ion: 864  Y-ion: 474  Z-ion: 1109 |
| 5 | 1345.97 | 1345.67 | Hex_3_HexNAc_2_Fuc_1_ | 9.74 |  | B-ions: 894, 1068  C-ions: 433, 667  Y-ion: 474 |
| 6 | 1376.00 | 1375.68 | Hex_4_HexNAc_2_ | 6.80 |  |  |
| 7 | 1387.03 | 1386.70 | Hex_2_HexNAc_3_Fuc_1_ | 0.91 |  |  |
| 8 | 1417.04 | 1416.71 | Hex_3_HexNAc_3_ | 2.86 |  |  |
| 9 | 1520.12 | 1519.76 | Hex_3_HexNAc_2_Fuc_2_ | 3.64 |   | B-ion: 1068  C-ion: 433  Y-ions: 474, 678, 1109 |
| 10 | 1550.13 | 1549.77 | Hex_4_HexNAc_2_Fuc_1_ | 3.32 | 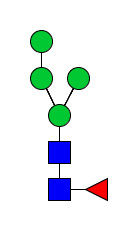 | B-ions: 894, 1098  C-ions: 433, 667  Y-ions: 474, 678 |
| 11 | 1580.13 | 1579.78 | Hex_5_HexNAc_2_ | 1.30 |  |  |
| 12 | 1591.16 | 1590.80 | Hex_3_HexNAc_3_Fuc_1_ | 4.62 |  |  |
| 13 | 1662.22 | 1661.84 | Hex_3_HexNAc_4_ | 0.70 |  | B-ion: 527  C-ions: 1402, 545  (or Y-ion)  Y-ions: 953, 1157 (or C-ion) |
| 14 | 1784.29 | 1783.88 | Hex_6_HexNAc_2_ | 3.43 |  |  |
| 15 | 1795.31 | 1794.90 | Hex_4_HexNAc_3_Fuc_1_ | 1.53 | 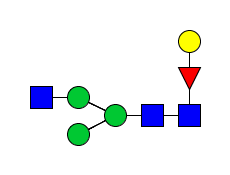 | B-ions: 1139, 486  C-ions: 433, 1157  Y-ion: 1331 |
| 16 | 1836.34 | 1835.92 | Hex_3_HexNAc_4_Fuc_1_ | 3.85 |   | B-ions: 701, 905  C-ion: 1331  Y-ions: 474, 1157 |
| 17 | 1907.14 | 1906.96 | Hex_3_HexNAc_5_ | 0.23 | 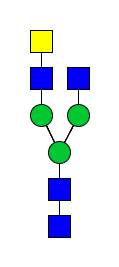 |  |
| 18 | 1988.44 | 1987.98 | Hex_7_HexNAc_2_ | 2.36 |  |  |
| 19 | 2010.46 | 2010.01 | Hex_3_HexNAc_4_Fuc_2_ | 5.51 |  | B-ions: 701 (or Z-ion), 905, 1558  Y-ions: 474, 1127, 1332 (or C-ion), 1751 |
| 20 | 2040.48 | 2040.02 | Hex_4_HexNAc_4_Fuc_1_ | 2.78 | 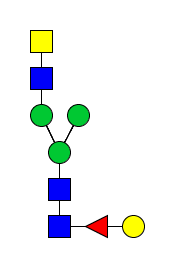 |  |
| 21 | 2081.52 |  | Hex_3_HexNAc_5_Fuc_1_ | 0.38 | 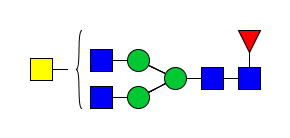 |  |
| 22 | 2151.54 |  | Hex_3_HexNAc_6_ | 0.52 | 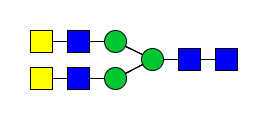 |  |
| 23 | 2192.58 | 2192.08 | Hex_8_HexNAc_2_ | 2.01 |  |  |
| 24 | 2214.60 | 2214.11 | Hex_4_HexNAc_4_Fuc_2_ | 3.79 |  | B-ion: 905 (or Z-ion)  C-ion: 433  Y-ions: 1331 (or C-ion), 1536 |
| 25 | 2244.63 | 2244.12 | Hex_5_HexNAc_4_Fuc_1_ | 0.69 | 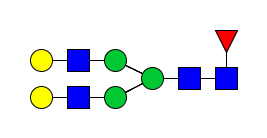 |  |
| 26 | 2255.64 | 2255.14 | Hex_3_HexNAc_5_Fuc_2_ | 1.15 | 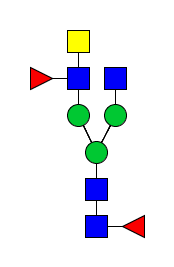 |  |
| 27 | 2388.73 | 2388.20 | Hex_4_HexNAc_4_Fuc_3_ | 0.78 | 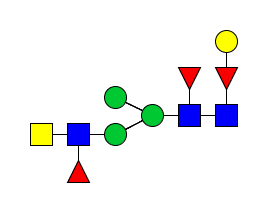 |  |
| 28 | 2396.70 | 2396.18 | Hex_9_HexNAc_2_ | 4.40 |  |  |
| 29 | 2418.75 | 2418.21 | Hex_5_HexNAc_4_Fuc_2_ | 0.47 | 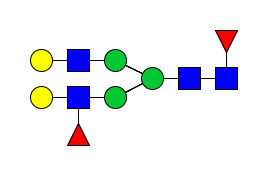 |  |
| 30 | 2459.78 | 2459.24 | Hex_4_HexNAc_5_Fuc_2_ | 0.80 | 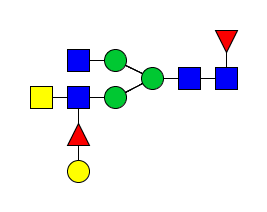 |  |
| 31 | 2500.81 | 2500.27 | Hex_3_HexNAc_6_Fuc_2_ | 0.33 | 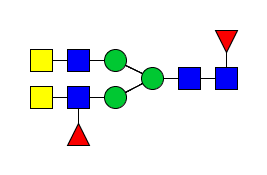 |  |
| 32 | 2600.85 | 2600.28 | Hex_10_HexNAc_2_ | 0.12 |  |  |
| 33 | 2633.90 | 2633.33 | Hex_4_HexNAc_5_Fuc_3_ | 0.13 | 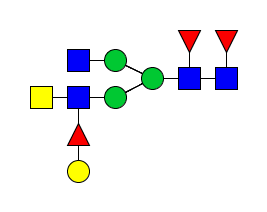 |  |
| 34 | 2663.93 | 2633.34 | Hex_5_HexNAc_5_Fuc_2_ | 0.17 | 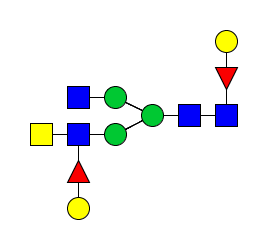 |  |
| 35 | 2674.94 | 2674.36 | Hex_3_HexNAc_6_Fuc_3_ | 0.79 | 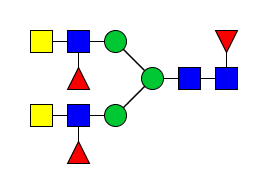 | See Figure S1A  B-ions: 905, 2223  C-ion: 1996  Y-ions: 474, 1996, 2415  Z-ion: 701  YZ-ion: 1095 (or CZ-ion) |
| 36 | 2704.95 | 2704.37 | Hex_4_HexNAc_6_Fuc_2_ | 0.19 | 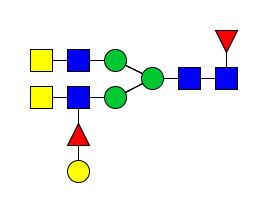 |  |
| 37 | 2879.08 | 2878.46 | Hex_4_HexNAc_6_Fuc_3_ | 0.45 | 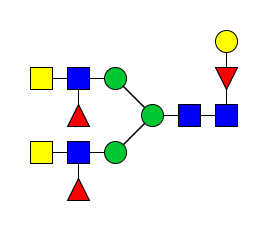 | See Figure S1B  B-ions: 701, 905  C-ion: 433  Y-ions: 1996 (or C-ion), 2200, 2620 |
| 38 | 3083.22 | 3082.56 | Hex_5_HexNAc_6_Fuc_3_ | 0.21 | 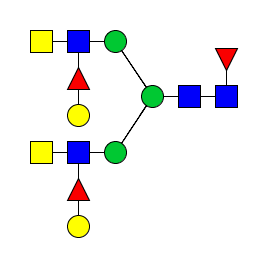 | See Figure S1C  B-ion: 905  C-ion: 433  Z-ion: 2672  Y-ions: 1996, 2200  BY-ion: 646 |

^a^ Calculated as (area of each peak/total area of all glycan peaks) × 100%.
