# Supplementary material for: H11-induced immunoprotection is predominantly linked to N-glycan moieties during Haemonchus contortus infection
Source: Front Immunol. 2022 Oct 25;13:1034820. doi: 10.3389/fimmu.2022.1034820 (PMC9667387; doi:10.3389/fimmu.2022.1034820)
Supplement: Supplementary Table 4 — N-glycan configurations predicted for native H11 from Haemonchus contortus released by PNGase A. [file Table_4.docx]

**SUPPLEMENTARY TABLE 4 |** N-glycan configurations predicted for native H11 from *Haemonchus contortus* released by PNGase A.

| **No.** | **Observed *m/z***  **[M + Na]^+^** | **Calculated *m/z* [M + Na]^+^** | **Composition** | **Relative**  **abundance (%)^a^** | **Proposed structures** | **Key MS/MS fragments**  ***m/z*** |
| --- | --- | --- | --- | --- | --- | --- |
| 1 | 1316.00 | 1315.66 | Hex_2_HexNAc_2_Fuc_2_ | 3.06 | 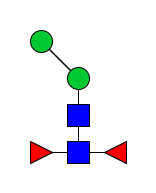 |  |
| 2 | 1346.02 | 1345.67 | Hex_3_HexNAc_2_Fuc_1_ | 15.22 | 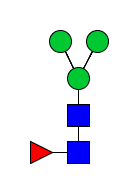 | B-ion: 894  C-ion: 667  Y-ion: 474 |
| 3 | 1376.04 | 1375.68 | Hex_4_HexNAc_2_ | 10.56 | 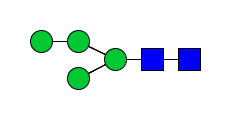 |  |
| 4 | 1417.08 | 1416.71 | Hex_3_HexNAc_3_ | 4.03 |  |  |
| 5 | 1520.15 | 1519.76 | Hex_3_HexNAc_2_Fuc_2_ | 9.06 | 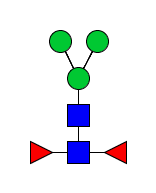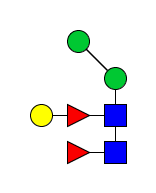 | B-ions: 463, 1068  C-ions: 433, 667  Y-ions: 474, 648  Z-ion: 1109 |
| 6 | 1550.17 | 1549.77 | Hex_4_HexNAc_2_Fuc_1_ | 2.78 | 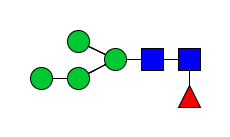 |  |
| 7 | 1580.18 | 1579.78 | Hex_5_HexNAc_2_ | 22.22 |  |  |
| 8 | 1591.20 | 1590.80 | Hex_3_HexNAc_3_Fuc_1_ | 5.30 | 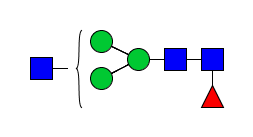 |  |
| 9 | 1694.28 | 1693.85 | Hex_3_HexNAc_2_Fuc_3_ | 1.24 | 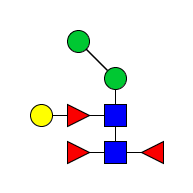 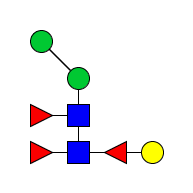 | B-ions: 445, 864, 1068  C-ions: 433, 463  Y-ions: 648, 1271  Z-ion: 835  BY-ion: 676 |
| 10 | 1724.31 | 1723.86 | Hex_4_HexNAc_2_Fuc_2_ | 0.78 | 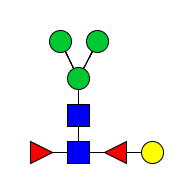 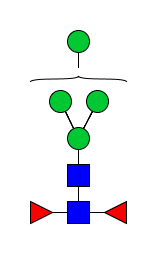 | B-ions: 894, 1098  C-ions: 433, 667, 1117  Y-ion: 893  Z-ion: 835, 1098, 1313, 1487 |
| 11 | 1784.33 | 1783.88 | Hex_6_HexNAc_2_ | 4.13 |  |  |
| 12 | 1836.39 | 1835.92 | Hex_3_HexNAc_4_Fuc_1_ | 3.38 | 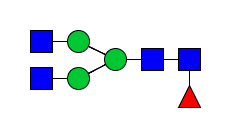 | B-ion: 1384  Y-ion: 474  Z-ions: 701, 1629 |
| 13 | 1988.48 | 1987.98 | Hex_7_HexNAc_2_ | 2.52 |  |  |
| 14 | 2010.51 | 2010.01 | Hex_3_HexNAc_4_Fuc_2_ | 4.18 | 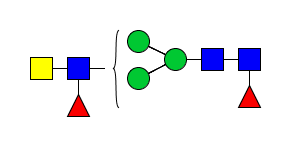 | B-ions: 701 (or Z-ion), 905, 1558  Y-ions: 474, 1127, 1332 (or C-ion), 1751 |
| 15 | 2040.53 | 2040.02 | Hex_4_HexNAc_4_Fuc_1_ | 1.82 | 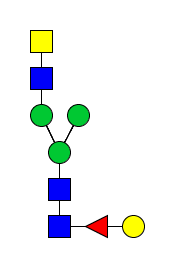 |  |
| 16 | 2192.62 | 2192.08 | Hex_8_HexNAc_2_ | 2.08 |  |  |
| 17 | 2214.65 | 2214.11 | Hex_4_HexNAc_4_Fuc_2_ | 1.98 |  | B-ion: 905 (or Z-ion)  C-ion: 433  Y-ions: 1331 (or C-ion), 1536 |
| 18 | 2396.76 | 2396.18 | Hex_9_HexNAc_2_ | 5.65 | (Man9) |  |

^a^ Calculated as (area of each peak)/(total area of all glycan peaks) × 100%.
