# Supplementary material for: H11-induced immunoprotection is predominantly linked to N-glycan moieties during Haemonchus contortus infection
Source: Front Immunol. 2022 Oct 25;13:1034820. doi: 10.3389/fimmu.2022.1034820 (PMC9667387; doi:10.3389/fimmu.2022.1034820)
Supplement: Supplementary Table 5 — List of aminopeptidases and their N-glycosylation sites identified in native H11 from Haemonchus contortus. [file Table_5.docx]

**SUPPLEMENTARY TABLE 5 |** List of aminopeptidases and their N-glycosylation sites identified in native H11 from *Haemonchus contortus.*

| **Protein accession** | **Protein description** | **Gene name** | **Position** | **Amino acid** | **Enzyme/s** | **No. of sites** |
| --- | --- | --- | --- | --- | --- | --- |
| Q9U5P5 | Aminopeptidase | *h11-2* | 549 | N | PNGase F | 1 |
| Q967C6 | Aminopeptidase | *h11-4* | 548 | N | PNGase F | 1 |
| V5K5H8 | Aminopeptidase | *h11-5* | 300 | N | PNGase F | 1 |
| A0A126UAR8 | Aminopeptidase | *ap-1* | 98 | N | PNGase A | 1 |
| A0A126UB22 | Aminopeptidase | *ap-5* | 121 | N | PNGase F | 6 |
|  |  |  | 229 | N | PNGase F |  |
|  |  |  | 305 | N | PNGases F and A |  |
|  |  |  | 771 | N | PNGases F and A |  |
|  |  |  | 860 | N | PNGase A |  |
|  |  |  | 95 | N | PNGase F |  |
| A0A140EQK0 | Aminopeptidase | *ap-11* | 148 | N | PNGase F | 3 |
|  |  |  | 285 | N | PNGases F and A |  |
|  |  |  | 318 | N | PNGases F and A |  |
